# A short pre-conception bout of predation risk affects both children and grandchildren

**DOI:** 10.1038/s41598-023-37455-9

**Published:** 2023-07-05

**Authors:** Sriya Bhattacharya, Phillip E. MacCallum, Mrunal Dayma, Andrea McGrath-Janes, Brianna King, Laura Dawson, Francis R. Bambico, Mark D. Berry, Qi Yuan, Gerard M. Martin, Evan L. Preisser, Jacqueline J. Blundell

**Affiliations:** 1 Department of Psychology, Memorial University of Newfoundland, St. John’s, NL A1B 3X9 Canada; 2 Department of Biochemistry, Memorial University of Newfoundland, St. John’s, NL A1B 3X9 Canada; 3 Biomedical Sciences, Faculty of Medicine, Memorial University of Newfoundland, St. John’s, NL A1B 3X9 Canada; 4 Department of Biological Sciences, University of Rhode Island, Kingston, RI 02881 USA; 5 Present Address: Northwestern Polytechnic, Grande Prairie, AB T8V 4C4 Canada

**Keywords:** Ecology, Neuroscience

## Abstract

Traumatic events that affect physiology and behavior in the current generation may also impact future generations. We demonstrate that an ecologically realistic degree of predation risk prior to conception causes lasting changes in the first filial (F1) and second filial (F2) generations. We exposed male and female mice to a live rat (predator stress) or control (non-predator) condition for 5 min. Ten days later, stressed males and females were bred together as were control males and females. Adult F1 offspring from preconception-stressed parents responded to a mild stressor with more anxiety-like behavior and hyperarousal than offspring from control parents. Exposing these F1 offspring to the mild stressor increased neuronal activity (cFOS) in the hippocampus and altered glucocorticoid system function peripherally (plasma corticosterone levels). Even without the mild stressor, F1 offspring from preconception-stressed parents still exhibited more anxiety-like behaviors than controls. Cross-fostering studies confirmed that preconception stress, not maternal social environment, determined offspring behavioral phenotype. The effects of preconception parental stress were also unexpectedly persistent and produced similar behavioral phenotypes in the F2 offspring. Our data illustrate that a surprisingly small amount of preconception predator stress alters the brain, physiology, and behavior of future generations. A better understanding of the ‘long shadow’ cast by fearful events is critical for understanding the adaptive costs and benefits of transgenerational plasticity. It also suggests the intriguing possibility that similar risk-induced changes are the rule rather than the exception in free-living organisms, and that such multigenerational impacts are as ubiquitous as they are cryptic.

## Introduction

Predators pose an existential threat to prey, and even individuals that survive predator encounters are often forever changed by the experience. While predator-risk-induced trait changes ‘RITRs’^1^, in behavior, physiology, and development reduce the risk of death, the long-term costs of such alterations can be substantial^2–4^. These costs have been documented in a wide array of both lab and field systems. Laboratory studies exposing organisms to predators or predator cues have documented lasting RITRs in social and anxiety-like behaviors, arousal, and impairments in learning and memory, with corresponding alterations in neuronal and hypothalamic–pituitary–adrenal (HPA) axis activity^5–11^. Analogous risk manipulations in free-living animals have documented similarly strong effects on prey neurobiology^12^ as well as growth and fitness^13–15^: exposing songbirds to recordings of predatory bird calls, for instance, reduced their fecundity by 40%^16^.

Our understanding of the importance of RITRs has been accompanied by increasing recognition that they can also cast a ‘long shadow’ that affects future generations^17–21^. Such transgenerational plasticity (‘TGP’), defined broadly as alterations to offspring phenotype in response to the parental environment^17^, can play an important role in preparing future generations for the challenges posed by a variable environment^22,23^. The idea that the effects of preconception stress can be seen in subsequent generation agrees with research documenting a higher incidence of psychiatric illness in the children and grandchildren of Holocaust survivors^24–27^. Both field and laboratory studies of these far-reaching predator impacts have documented behavioral, physiological, and morphological changes in the future offspring of stressed prey animals. The offspring of freshwater snails exposed to predator cues have altered anti-predator behaviors^28^ and harder-to-crush shells^29^ than control offspring, for instance, while the progeny of predator-stressed snowshoe hares have higher stress levels^30^. Prenatal predator stress in mammals can decrease the gestational length of pregnancy, litter size, and pre-weaning survival rate^4,31–34^, and surviving pups often show altered developmental trajectories e.g., body weight, seizure susceptibility;^33,35–37^. Offspring of mothers exposed to predator stress during pregnancy display increased predator avoidance, altered sociability, learning and memory impairments, novelty-induced anxiety, and increased corticosterone levels^4,38–41^. These effects are not confined to mammalian, or even vertebrate, systems; exposure to predator cues alters offspring telomere length in pied flycatchers^42^ and anti-predator behavior in the offspring of both crickets^43^ and marine snails^44^.

It is increasingly clear that predator stress prior to pregnancy also has the potential to affect offspring reviewed in^21^. The ecological implications of such changes are profound since it extends the ‘window’ for stress exposure effects well beyond the short period of pregnancy. Increasing the period during which parental stressors can affect future generations may improve the likelihood of producing offspring suited to their environment, a major benefit of TGP^45,46^. Preconception maternal exposure to predator cues altered F1 offspring phenotype in several water flea species^47,48^ and both F1 and F2 offspring in rotifers^49^. In mammals, Dias and Ressler^50^ demonstrated that odor fear conditioning of male F0 mice ten days prior to mating increased the sensitivity of naïve F1 progeny to that odor. Subsequent research into preconception predator stress found that paternal exposure to an artificial predator odor altered antipredator behavior in F1 mice^51^. Similarly, exposing both male and female rats to chronic preconception cat exposure (2 h/day for 15–50 days) increased epileptic behaviors and anxiogenic responses in their offspring^52–54^.

While preconception predator stress clearly has the potential to affect F1 offspring, whether it can also shape individuals in the F2 generation and beyond is largely unexplored but see^55^. If so, the ‘long shadows’ cast by preconception predator encounters could exert profound but cryptic effects on current-day species interactions. Research addressing this question is challenging because of the tightly controlled conditions necessary to isolate the stressor signal from background environmental variation e.g.^19^. Although such precise manipulations generally require laboratory-based work, their results inform our understanding of free-living organisms only if the timing and duration of the preconception predator stressor is ecologically realistic^56^. Few free-living animals, for instance, would survive hours of close-proximity exposure to a predator^52–54^. While such changes generally benefit future generations^38,46,57^, they may also be maladaptive^21^; understanding when such effects occur and how they manifest themselves is critical to improving our understanding of transgenerational plasticity.

We describe research exploring whether an ecologically realistic degree of parental predation risk (a single short bout of preconception stress) alters the brain and behavior of both F1 and F2 offspring. Adolescent F1 offspring from predator-stressed or control parents underwent an extensive behavioral battery to assess anxiety- and depression-like behaviors, arousal, and social behavior. These behaviors were also assessed following a mild psychogenic stressor in adult F1s to determine if parental experience altered offspring stress sensitivity. We similarly examined F2 individuals to determine the longevity of intergenerational predator-stress-induced behavioral changes. A cross-fostering study was conducted to determine if F1 behavioral changes were due to the maternal social environment. Finally, in both the adult and F1 generation, we also assessed HPA axis (plasma corticosterone) activity and neural activation (cFOS expression) in the hippocampus, a brain area known to be involved in the stress response. Our results demonstrate that acute parental exposure to predation risk engenders lasting effects on multiple subsequent generations, a result that suggests the intriguing possibility that similar transgenerational responses are the rule rather than the exception in free-living organisms. These findings may improve our understanding of the etiology of stress-related psychopathologies such as post-traumatic stress, anxiety, and mood disorders.

## Results

### Five-minute rat exposure increased anxiety-like behavior, elevated corticosterone, and increased cFOS expression in the hippocampus

**Figure 1:**
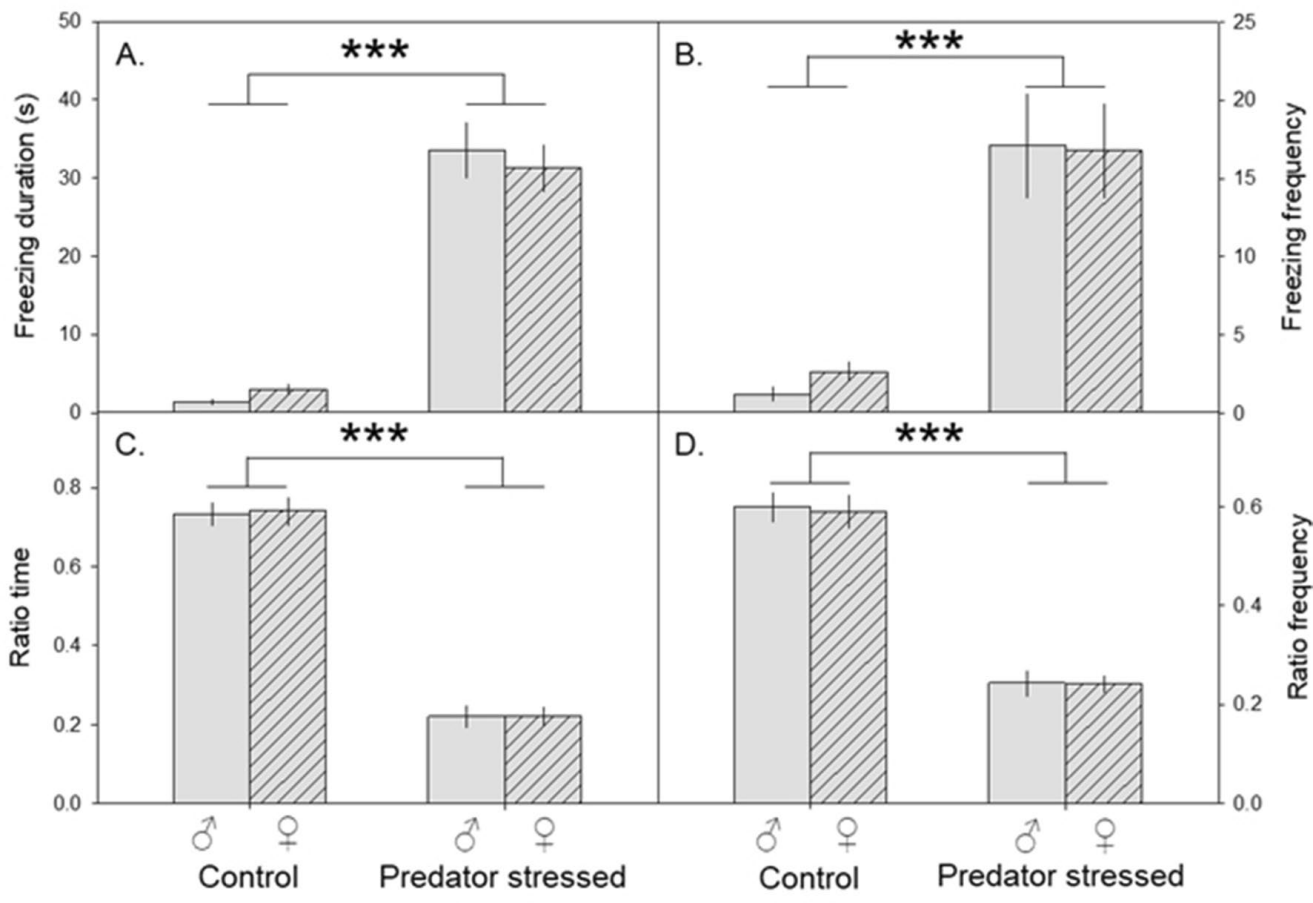
Five-minute rat exposure increased anxiety-like behavior. (**A,B**) Mean ± SEM is plotted for freezing duration and frequency. Mice exposed to a rat for 5 min froze for longer (**A**) and more often (**B**) than mice exposed to an empty cage (control mice). (**C,D**) Mean ± SEM for activity in the elevated plus maze. Predator-stressed mice also spent less time in the open arms (**C**) and entered the open arms less often (**D**) than control mice.

After male and female mice were subject to either a five-minute rat exposure or control conditions, we assessed anxiety-like behavior, plasma corticosterone, and cFOS expression in the hippocampus. Mice exposed to a rat (predator stressed) for five minutes froze for longer (L-R χ^2^_1 df_ = 90.8, p < 0.0001, Fig. 1A) and more often (χ^2^_1 df_ = 32.5, p < 0.0001, Fig. 1B) than mice exposed to an empty cage (control mice). Two days later when tested in the elevated plus maze (EPM), predator-stressed mice also spent less time in the open arms (ratio time, χ^2^_1 df_ = 132, p < 0.0001, Fig. 1C) and entered the open arms less often (ratio frequency, χ^2^_1 df_ = 95.3, p < 0.0001, Fig. 1D) than control mice. There was no significant effect of sex or any two-way interactions in the exposure or EPM (all p > 0.05). In addition, predator-stressed mice displayed higher plasma corticosterone levels than controls (L-R χ^2^_1 df_ = 9.73, p = 0.018, Suppl Fig. 1). Finally, predator stress increased c-FOS expression in the hippocampus relative to unstressed controls. As shown in Suppl Fig. 2, predator stress increased c-FOS expression in the dentate gyrus (dorsal left hemisphere ‘DENTLH’, L-R χ^2^_1 df_ = 23.2, p < 0.0001; dorsal right hemisphere ‘DENTRH’, χ^2^_1 df_ = 34.0, p < 0.0001; ventral left hemisphere ‘VDLH’, χ^2^_1 df_ = 23.5, p < 0.0001; ventral right hemisphere ‘VDRH’, χ^2^_1 df_ = 5.36, p = 0.0206) and CA1 (dorsal right hemisphere ‘CA1RH’, χ^2^_1 df_ = 20.3, p < 0.0001; ventral left hemisphere ‘VCA1LH’, χ^2^_1 df_ = 9.13, p = 0.0025; ventral right hemisphere VCA1RH χ^2^_1 df_ = 20.4, p < 0.0001) compared to controls. There was no effect of treatment on the dorsal left hemisphere (‘CA1LH’, χ^2^_1 df_ = 0.11, p = 0.73).

### The effects of parental preconception predator stress on F1 mice

**Figure 2:**
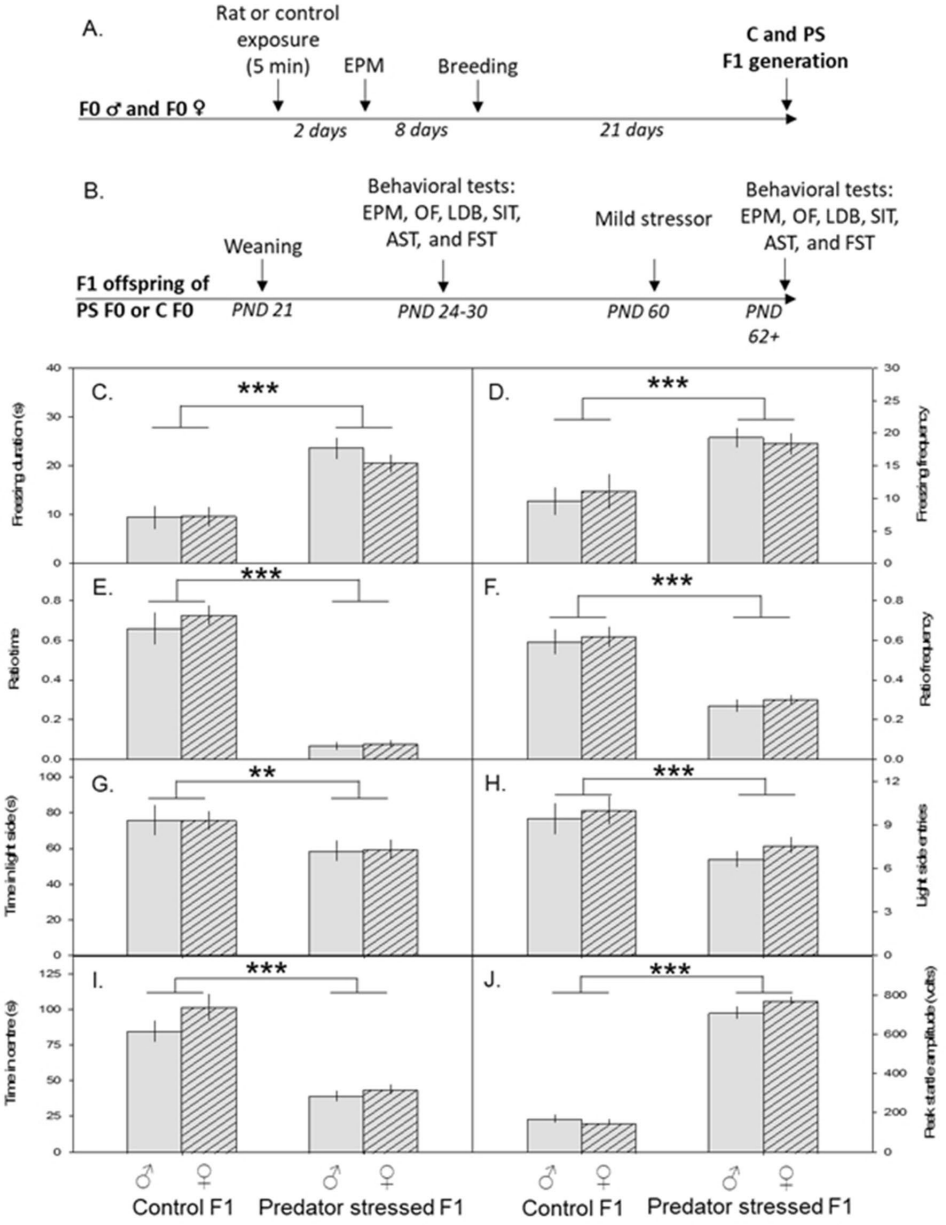
Pre-conception predator stress produces anxiety-like behaviour in first filial (F1) mice. (**A**) Schematic of F0 procedure. (**B**) Schematic of the F1 procedure. (**C–J**) Mean ± SEM plotted over four groups: male and female offspring from control parents (control F1♂ and ♀), or male and female offspring from preconception predator-stressed parents (predator stress F1♂ and ♀). F1 offspring of predator-stressed parents froze longer (**C**) and more often (**D**) than control F1s during the mild stressor (2 min rat exposure). Following the mild stressor, predator stressed F1s spent less time in the open arms (ratio time, (**E**)) and entered the open arms less often (ratio frequency, (**F**)) in the elevated plus maze, spent less time (**G**) and entered (**H**) the light side less often in the light/dark box, spent less time in the center (**I**) of the open field, and had an increased peak startle amplitude (**J**) compared to control F1s. *EPM* elevated plus maze, *OF* open field, *LDB* light/dark box, *SIT* social interaction test, *FST* forced swim test, *AST* acoustic startle test, *PS* predator stress, *C* control. **p < 0.01, ***p < 0.001.

A subset of the mice described in “Five-minute rat exposure increased anxiety-like behavior, elevated corticosterone, and increased cFOS expression in the hippocampus” were bred with each other (stressed males with stressed females, control males with control females, Fig. 2A) and we examined behavior in adolescent F1s (Fig. 2B). In adulthood, all F1s were exposed to a mild stressor (2-min rat exposure) and behavior (Fig. 2B), plasma corticosterone, and cFOS expression in the hippocampus were assessed. The 2-min rat exposure was considered ‘mild’ as it did not produce significant changes in anxiety-like behaviors in naïve mice (supplementary methods 1.4, supplementary results 2.1).

#### Parental preconception predator stress increases anxiety-like behaviour and hyperarousal

In adolescence, F1s from preconception-stressed parents avoided the EPM open arms (ratio time: F_1,14.1_ = 37.9, p < 0.0001; ratio frequency: F_1,15.4_ = 17.8, p = 0.0007; Suppl Fig. 3A, B), spent less time in the center and travelled a shorter distance in the open field (OF; F_1,13.8_ = 14.6, p = 0.0019; F_1,13.3_ = 29.2, p < 0.0001; Suppl Fig. 3C,D), interacted less with a social target in the social interaction test (SIT; F_1,8.88_ = 8.06, p = 0.020; Suppl Fig. 3E), and had a stronger response in the acoustic startle test (AST; peak startle amplitude: F_1,15.6_ = 38.1, p < 0.0001; Suppl Fig. 3F) than offspring from control parents. There was no significant effect of sex nor any significant interactions on any behavioral measure (all p > 0.05).

In adulthood, all F1s were exposed to a mild stressor (2-min rat exposure). During the 2 min rat exposure, F1s from preconception-stressed parents froze longer (F_1,98.01_ = 38.2, p < 0.0001) and more often (F_1,98_ = 50.8, p < 0.0001) than F1s from control parents (Fig. 2C,D). After the exposure, F1s from preconception-stressed parents also avoided the open arms of the EPM (ratio time: F_1,98.02_ = 258, p < 0.0001; ratio frequency: F_1,98.1_ = 60.6, p < 0.0001; Fig. 2E,F), spent less time in the light side of the LDB (light side duration: F_1,98.01_ = 10.5, p = 0.002; light side entries F_1,98.01_ = 20.5, p < 0.0001; Fig. 2G,H), spent less time in the center (F_1,98_ = 74.1, p < 0.0001, Fig. 2I) and travelled less (F_1,97.21_ = 42.9, p < 0.0001) in the OF, and had a stronger startle response in the AST (peak startle amplitude: F_1,99_ = 474, p < 0.0001; Fig. 2J) than F1s from control parents. There was no effect of treatment on time spent immobile in the FST or on the social interaction ratio (both p < 0.10), and no significant effect of sex (all p > 0.05) on any of the variables. These results demonstrate that parental preconception stress generally makes adult F1s more sensitive to a mild stressor.

We used a separate set of F1s to determine if the preconception-stressed behavioral phenotype in the F1 persisted into adulthood *in the absence of the mild stressor.* Consistent with our previous results, adult F1s from stressed parents avoided the open arms of the EPM (ratio time: F_1,4.31_ = 37.6, p = 0.0028; ratio frequency: F_1,6.13_ = 14.4, p = 0.0087; Suppl Fig. 5A, B), travelled less distance (F_1,8.45_ = 19.4, p = 0.002; Suppl Fig. 5C) and spent marginally less time in the center (F_1,1_ = 157, p = 0.0506; Suppl Fig. 5D) of the OF. They also spent less time in the light side of the LDB (F_1,4.68_ = 15.5, p = 0.0125; Suppl Fig. 5E) and had a higher startle (peak startle amplitude: F_1,4.16_ = 10.3, p = 0.0307; Suppl Fig. 5F) than F1s from control parents. There were no other significant differences across groups or sex differences (all p > 0.05). Our results demonstrate that parental preconception stress affects adult F1 behavior in the absence of a stressful trigger event.

#### Elevated plasma corticosterone levels following a stressor (Fig. 3A)

**Figure 3:**
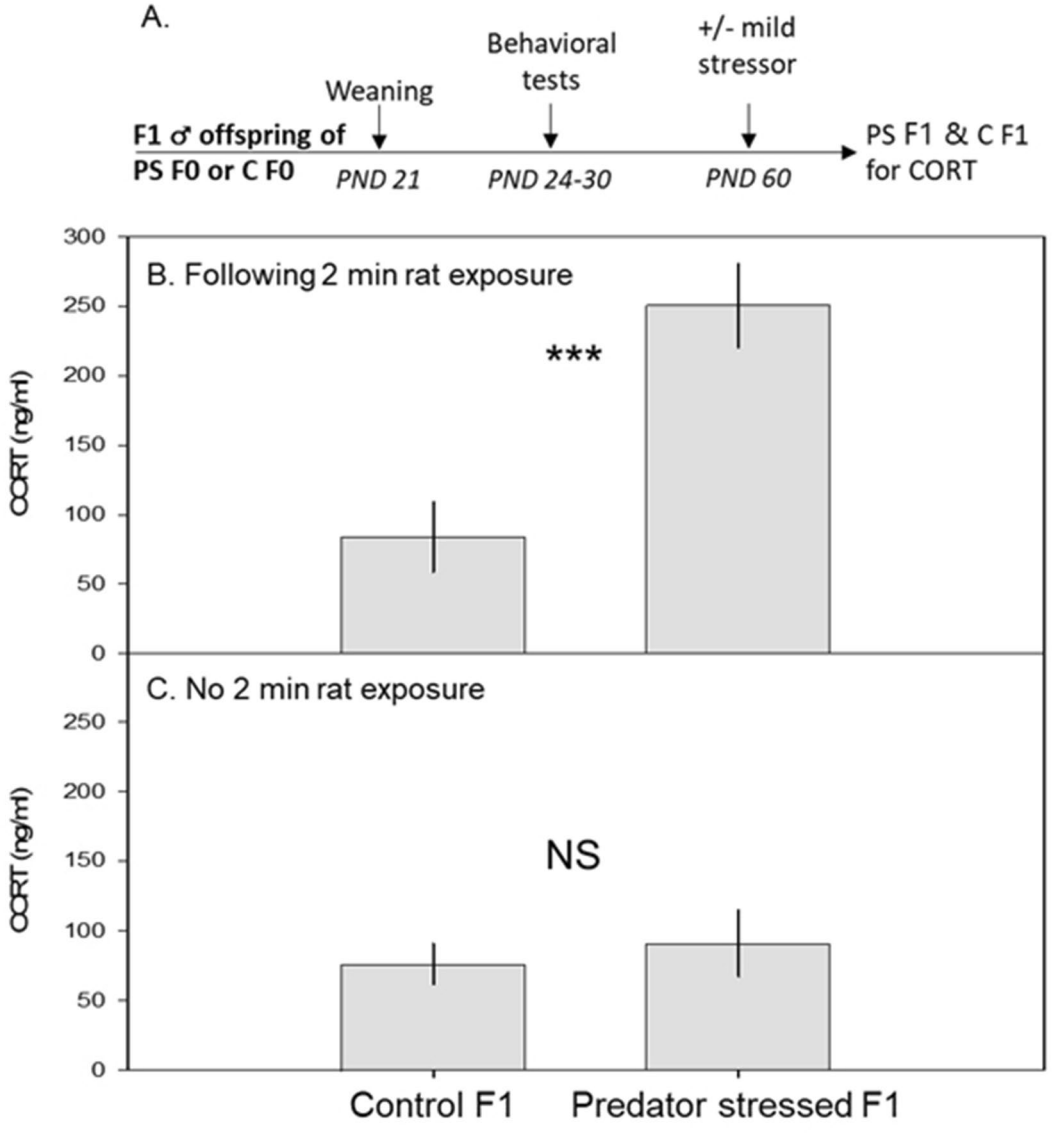
Pre-conception predator stress increases plasma corticosterone levels after a mild stressor in F1 mice. (**A**) Schematic of F1 corticosterone procedure. (**B,C**) Mean ± SEM plasma corticosterone (cort) in ng/ml plotted for two groups of offspring: those from control parents (control F1) and those from preconception predator-stressed parents (predator stress F1). Following the mild stressor, predator stressed F1s had increased serum cort levels compared to control F1s (**B**). In the absence of mild stressor, plasma cort levels did not differ across groups (**C**). *PS* predator stress, *C* control. ***p < 0.001, *NS* not significant.

Following the mild stressor (two-minute rat exposure), F1s from preconception-stressed parents had higher plasma corticosterone levels than F1s from control parents (L-R χ^2^_1 df_ = 11.5, p = 0.0007; Fig. 3B). In the absence of this mild stressor, there were no between-group differences in plasma corticosterone levels (χ^2^_1 df_ = 0.34, p = 0.56; Fig. 3C) in another set of F1s.

#### Increased c-FOS activation in several brain regions following a stressor

**Figure 4:**
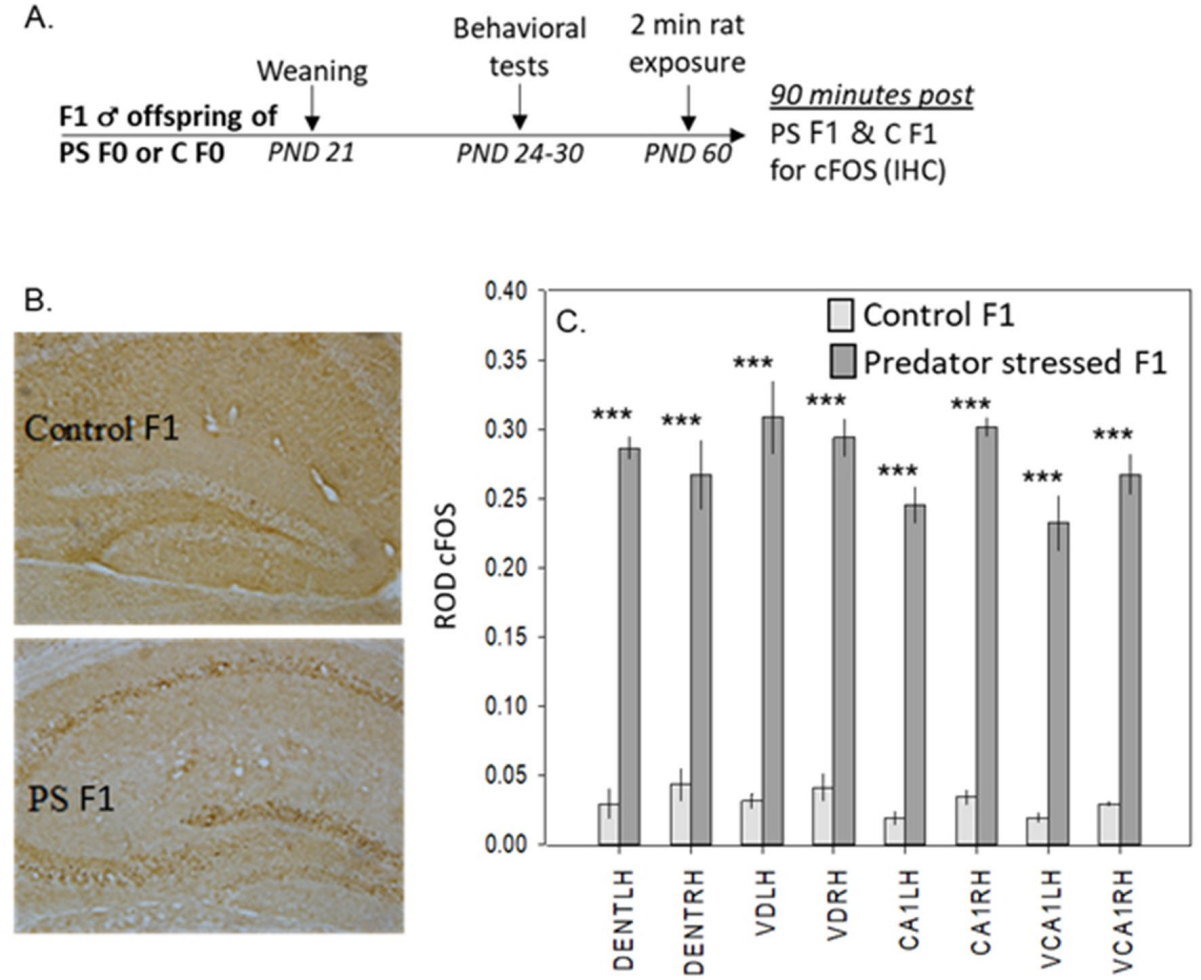
Pre-conception predator stress increases c-FOS expression after a mild stressor in F1 mice. (**A**) Schematic of F1 cFOS procedure. (**B**) Representative cFOS images in the dorsal hippocampus in offspring from either preconception predator-stressed parents (PS F1) or control parents (control F1). Expression of cFOS was measured in the hippocampus within the dorsal dentate gyrus, left and right hemisphere (DENTRH, DENTLH), ventral dentate gyrus, left and right hemisphere (VDLH, VDRH), dorsal CA1, left and right hemisphere (CA1LH, CA1RH), and ventral CA1, left and right hemisphere (VCA1LH, VCA1RH). (**C**) Mean ± SEM of cFOS in different brain regions. Following the mild stressor, offspring from predator stressed parents show elevated cFOS in the dentate and CA1 (ventral and dorsal, both hemispheres) compared to offspring from control parents. *C* control, *PS* predator stress. ***p < 0.001.

Following the mild stressor, preconception-stressed F1s had higher c-FOS expression in the dentate gyrus (DENTLH, L-R χ^2^_1 df_ = 33.9, p < 0.0001; DENTRH, χ^2^_1 df_ = 20.1, p < 0.0001; VDLH, χ^2^_1 df_ = 23.6, p < 0.0001; VDRH, χ^2^_1 df_ = 29.5, p < 0.0001) and CA1 (CA1LH, χ^2^_1 df_ = 30.8, p < 0.0001; CA1RH, χ^2^_1 df_ = 41.6, p < 0.0001; VCA1LH, χ^2^_1 df_ = 23.9, p < 0.0001; VCA1RH χ^2^_1 df_ = 30.4, p < 0.0001) than F1s from control parents (Fig. 4A–C).

#### Biological parent stress experience, not maternal social environment, determines anxiety-like behaviour and hyperarousal in F1 mice

**Figure 5:**
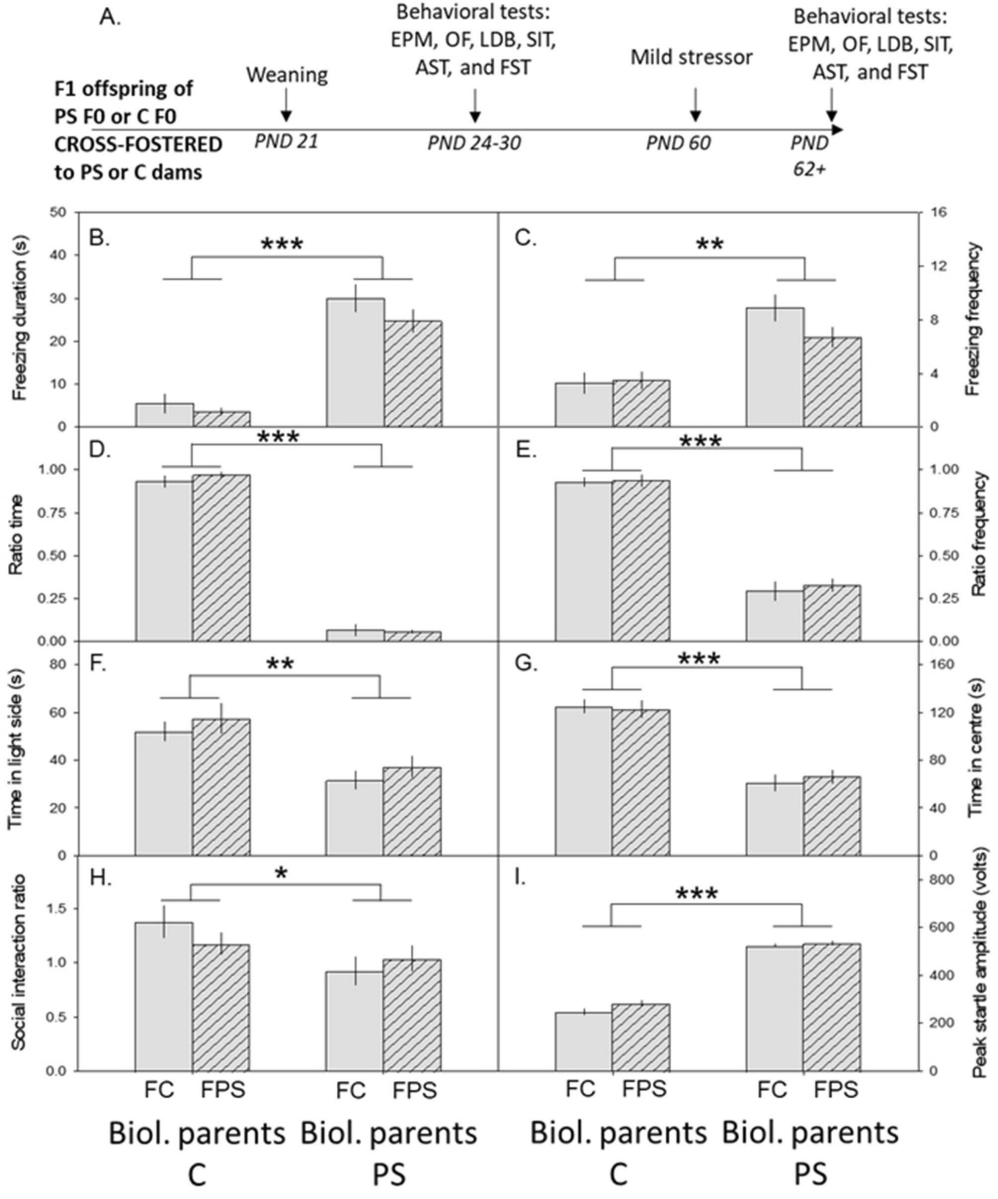
Biological parent stress experience, and not social environment, determines anxiety-like behaviour in F1 mice. (**A**) Schematic of the F1 procedure. (**B–I**) Mean ± SEM plotted over four groups: offspring of control biological parents that were cross-fostered to a control mother, offspring of control biological parents that were cross-fostered to a predator-stressed mother, offspring of predator-stressed biological parents that were cross-fostered to a control mother, and offspring of predator-stressed biological parents that were cross-fostered to a predator-stressed mother. During the mild stressor, the offspring of predator-stressed biological parents, regardless of their cross-fostering mother (predator stressed or control), froze longer (**B**) and more often (**C**) than offspring whose biological parents were controls. Following the mild stressor, offspring of predator-stressed biological parents, regardless of cross-fostering mother, spent less time in the open arms (ratio time, (**D**)) and entered the open arms less often (ratio frequency, (**E**)) in the elevated plus maze, spent less time (**F**) in the light/dark box, spent less time in the center (**G**) in the open field, spent less time with the social target (social interaction ratio, (**H**)) in the social interaction test, and had increased peak startle amplitude (**I**) compared to the offspring of control biological parents. *EPM* elevated plus maze, *OF* open field, *LDB* light/dark box, *SIT* social interaction test, *FST* forced swim test, *PS* predator stress, *C* control. *p < 0.05, **p < 0.01, ***p < 0.001.

We assessed anxiety- and depressive-like behaviours, social behaviour, and hyperarousal in adolescent F1 mice that had been cross-fostered (Fig. 5A). There was a significant main effect of biological parent in the EPM (ratio time: F_1,16.7_ = 58.1, p < 0.0001; ratio frequency: F_1,16.7_ = 58.1, p < 0.0001; Suppl Fig. 5A, B), LDB (light side entries: F_1,8.39_ = 5.32, p = 0.048; Suppl Fig. 5C), OF (distance travelled: F_1,11.7_ = 5.2, p = 0.042; Suppl Fig. 5D, E), and AST (peak startle amplitude: F_1,12.4_ = 5.86, p = 0.032; Suppl Fig. 5F). There was a small, but a significant main effect of foster parent, on time in center in the OF (F_1,10.4_ = 5.22, p = 0.045).

In adulthood, when these cross-fostered F1 offspring experienced a two-minute rat exposure (Fig. 5A), there was a significant main effect of biological parent on freezing (duration: F_1,10.1_ = 61.0, p < 0.0001; frequency: F_1,12.9_ = 29.2, p = 0.0043; Fig. 5B,C). Following the mild stressor, there was a significant main effect of biological parent in the EPM (ratio time: F_1,10.3_ = 1352, p < 0.0001; ratio frequency: F_1,11.8_ = 236, p < 0.0001, Fig. 5D,E), LDB (light side duration: F_1,10.6_ = 11.8, p = 0.0059, Fig. 5F), OF (time in center: F_1,10.3_ = 70.7, p < 0.0001, Fig. 5G), SIT (social interaction ratio: F_1,13.5_ = 8.11, p = 0.0133, Fig. 5H), and AST (peak startle amplitude: F_1,13.0_ = 677, p < 0.0001, Fig. 5I). There was also an effect of foster mother on the AST (individuals reared by stressed foster mothers had higher values; F_1,12.8_ = 4.89, p = 0.046) and an effect of offspring sex on the SIT (female offspring had higher values; F_1,105.6_ = 5.25, p = 0.024). These results suggest that experience of the biological parents largely drives the F1 behavioral phenotype, although the social environment plays a role in some behaviors.

Mother–pup behaviour was recorded for 40 min on alternate days from post-natal (PND) day 5–12. We also measured litter size, numbers of male and female pups, and percent of male pup’s outcomes for the cross-fostering F1 generation. Across all measures, there was no difference across groups (all p > 0.05). While not exhaustive, these data do suggest that regardless of pre-conception experience, mothers treated offspring similarly. Note also that all foster mothers accepted the new pups.

### Preconception predator stress-induced changes in F2 mice

**Figure 6:**
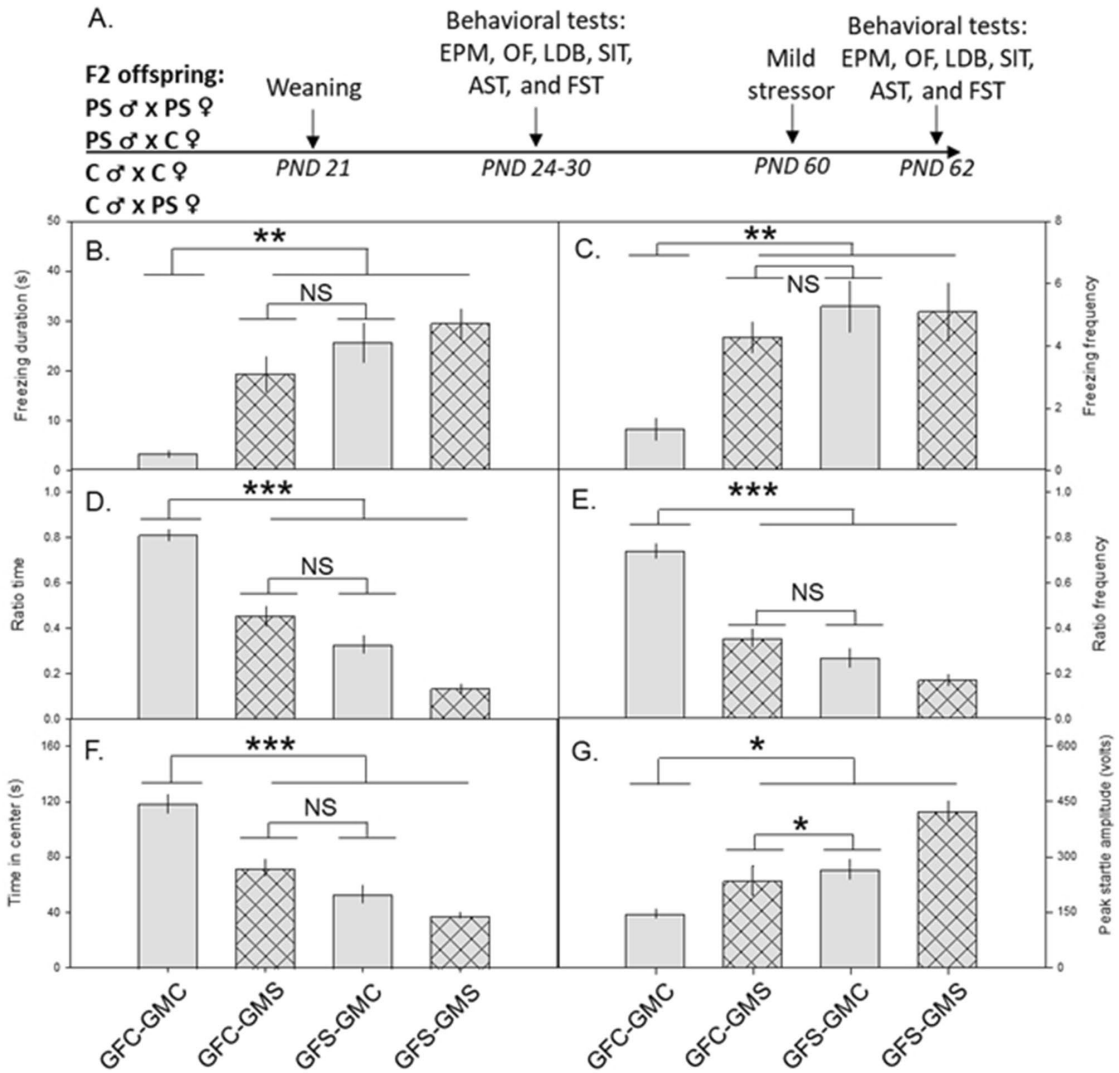
Preconception predator stress increased anxiety-like behaviour in second filial (F2) mice. (**A**) Schematic of the F2 procedure. (**B–G**) Mean ± SEM plotted over four groups: two control grandparents (GFC-GMC), control grandfather and predator-stressed grandmother (GFC-GMS), predator-stressed grandfather and control grandmother (GFS-GMC), and two predator-stressed grandparents (GFS-GMS). During the mild stressor, F2s with one or more predator-stressed grandparents froze longer (**B**) and more often (**C**) than F2s from control grandparents. Following the mild stressor, F2s with one or more predator-stressed grandparents spent less time in the open arms (ratio time, (**D**)) and entered the open arms less often (ratio frequency, (**E**)) in the elevated plus maze, spent less time in the center (**F**) in the open field, and had increased peak startle amplitude (**G**) compared to F2s from control grandparents. *EPM* elevated plus maze, *OF* open field, *LDB* light/dark box, *SIT* social interaction test, *FST* forced swim test, *PS* predator stress, *C* control. *p < 0.05, **p < 0.01, ***p < 0.001, *NS* not significant.

We assessed anxiety-like behaviour, hyperarousal, social interaction, and depressive-like behaviours in F2 mice. Control or predator-stressed F0s were bred with each other as described above to generate F1s. These F1s were reared to adulthood in the absence of any stressors and then used to generate four groups of F2 mice (GFC: grandfather control, GMC: grandmother control, GFS: grandfather predator-stressed, GMS: grandmother predator-stressed). Behavior of the four F2 groups (GFC-GMC, GFC-GMS, GFS-GMC, and GFS-GMS) was assessed during adolescence and again, following a mild stressor (two-minute rat exposure), in adulthood (Fig. 6A).

#### Grandparental preconception stress alters F2 behavior

To determine if there was a grandparental stress effect on adolescent F2s, planned comparisons were done comparing all three stressed groups (GFC-GMS, GFS-GMC and GFS-GMS) to the control group (GFC-GMC) across all behavioural measures. In the EPM, control F2s spent more time in the open arms (F_1,8.00_ = 25.7, p = 0.0010, Suppl Fig. 7A) and entered the open arms more often (F_1,8.89_ = 23.0, p = 0.0010, Suppl Fig. 7B) than the F2s from the three stressed groups. Control F2s also travelled more in the OF (F_1,8.03_ = 6.47, p = 0.0344, Suppl Fig. 7C) and spent more time in the center of the OF (F_1,7.33_ = 12.6, p = 0.0087, Suppl Fig. 7D) than F2s from the three stressed groups.

To determine whether there was a differential contribution from the grandmother and the grandfather to the adolescent F2 behavioural phenotype, planned comparisons were done comparing the GFS-GMC group to the GFC-GMS group. The GFS-GMC and GFC-GMS groups did not differ significantly in any of the measured variables (all p > 0.05).

In adulthood, all F2s were exposed to a mild stressor (two-minute rat exposure) (Fig. 6A). To determine if there was a grandparental stress effect, planned comparisons were done comparing the mean of all three stressed groups to the control group across all behavioural measures. F2s with at least one stressed grandparent displayed increased freezing behavior during the mild stressor than control F2s (freezing duration: F_1,7.66_ = 18.4, p = 0.0029; freezing frequency: F_1,5.89_ = 22.6, p = 0.0033, Fig. 6B,C). In the EPM, control F2s spent more time in the open arms (F_1,8.27_ = 32.8, p = 0.0004) and entered the open arms (F_1,8.04_ = 38.7, p = 0.0002) more often (Fig. 6D,E). They also spent more time in the center (F_1,7.66_ = 34.0, p = 0.0005; Fig. 6F) and travelled a greater distance in the OF (F_1,8.09_ = 12.1, p = 0.0081), and had a lower startle response in the AST (peak startle amplitude: F_1,8.70_ = 9.66, p = 0.0131, Fig. 6G) than F2s from the three stressed groups.

To determine whether there was a differential contribution from the grandmother and the grandfather to the adult F2 behavioural phenotype following the mild stressor, planned comparisons were done comparing the GFS-GMC group to the GFC-GMS group. Mean peak startle amplitude was higher in the GFS-GMC group than the GFC-GMS group (F_1,30_ = 5.66, p = 0.0239; Fig. 6G); the effect of treatment on all other variables was not significant (all p > 0.05).

## Discussion

Our results demonstrate that an ecologically realistic degree of predation risk—a single five-minute exposure to a predator—prior to conception engenders lasting effects on multiple subsequent generations. Such risk-induced trait responses (‘RITRs’) have been observed in a wide variety of species and potentially benefit both current and future generations via a reduced likelihood of damage or death from predation. The costs of such changes can manifest either immediately or over longer time scales. Laboratory experiments have documented that predation risk can alter both neural and HPA axis activity and cause lasting shifts in learning, memory, and behavior^58^, and analogous work with free-living prey species has found similar neurological impacts and significant effects on growth and fitness^59^. Despite debate over whether RITRs generally yield population-level effects^60,61^, there are well-documented cases where they do: exposing songbird populations to risk cues over multiple generations halved the number of juvenile recruits and drove the population into rapid decline^62^. Although the logistical challenges posed by such research are considerable, identifying the potential for, and mechanistic basis of, such long-term changes is essential for understanding their possible ecological impacts.

The fact that effects of transitory pre-conception predator exposure in the F0 generation were detectable in F1 and F2 offspring illustrates that even moderate predation risk can affect the neurobiology, physiology, and behavior of future generations^56^. We found that adult offspring from preconception-stressed parents were more responsive to a mild stressor than offspring from control (non-stressed) parents. The ‘parental stress’ F1s froze more during the stressor and afterwards had higher plasma corticosterone levels and increased cFOS expression in the hippocampus. In addition, ‘parental stress’ F1s showed increased anxiety-like behavior and hyperarousal during the week following the mild stressor. This is striking because the mild stressor we used (a 2 min rat exposure) did not alter behavior in our naïve mice. This suggests parental experience can alter the behavior of their offspring and neural activity in the hippocampus^58^. In the absence of the mild stressor, adolescent and adult behavior of ‘parental stress’ F1s was similar but somewhat less robust. Similar effects of transgenerational plasticity (TGP) on F1 individuals have been noted in a variety of systems^63^. The children of individuals suffering from post-traumatic stress disorder (PTSD), for instance, are more likely to diagnosed with PTSD or similar psychiatric conditions^64,65^, and the Holocaust has also affected the children and grandchildren of survivors^24–27^. Research on TGP in other mammal species found that parental exposure to predation risk can increase pre-weaning mortality and alter the development, behavior, and neurobiology of surviving F1 offspring^21^. In damselfish, parental exposure to cues from one predator species increased embryonic responses to cues from that predator but not to the cues from a novel predator species^46^. Importantly, TGP is not confined to vertebrate taxa^63^: parental exposure to predator cues alters F1 anti-predator behavior in crickets^43^ and several snail species^28,44^.

As a first step in identifying the neural mechanisms underlying this behavioral phenotype, we assessed cFOS expression in the hippocampus following a mild stressor in our F1 generation. We chose the hippocampus for its central role in consolidation of fear memories^66,67^, as well as its responsiveness to predator cues in wild animals^12^. We show that offspring from preconception predator stressed mice show increased c-FOS expression in the dentate gyrus and CA1 of the hippocampus following a mild stressor. It is not surprising that we see changes in neural activation in brain areas known to be involved in the stress response in the F0 generation; these mice were exposed to a stressor that was sufficient to produce lasting changes in anxiety-like behavior. In the F1, we exposed the mice to a 2 min RET, a stressor that does not alter behavior in naïve mice. Nevertheless, in response to the mild stressor, offspring from preconception stress mice show robust alterations in cFOS expression in stress-related brain areas. Our data suggest that the experience of the parents not only alters behavior of the offspring, but also neural activation. To our knowledge, this is the first demonstration that a mild stressor induces cFOS expression in the hippocampus in offspring from preconception predator-stressed parents. The data suggest that alterations in hippocampus (notably in the dorsal CA1 area^68^) may, at least initially, be important in the transmission of stress across generations. Future studies will include an examination of other brain areas relevant to the stress response including the paraventricular nucleus, amygdala and periaqueductal grey.

In species that exhibit parental care, parent-driven shifts in offspring phenotypes can arise from both epigenetic mechanisms and risk-induced changes in adult behaviour and/or other elements of the pre-weaning environment^17,20,50,69^. We assessed the relative contributions of maternal social environment and parental experience to TGP with a cross-fostering experiment in which the offspring of predator-stressed or control parents were reared by either predator-stressed or control foster mothers. Generally, regardless of foster mother condition, the adolescent offspring of predator-stressed biological parents exhibited more anxiety-like behavior and hyperarousal than the offspring of control biological parents. As adults, the offspring of predator-stressed biological parents responded more strongly to a mild stressor and exhibited increased anxiety-like behavior. These findings support the hypothesis that epigenetic changes caused by parental predation risk likely underlie the phenotypic shifts in F1 offspring and agree with previous studies e.g.,^50,70^ in the same model system that found F1 behaviour resulting from epigenetic changes rather than social transmission from the F0 generation. Despite the ubiquitous nature of the ‘biological parent’ effect in the current study, we did find a ‘foster parent’ effect on specific behaviors (e.g., time in the center of the OF) and hence, maternal social environment cannot be discounted when examining offspring from predator stressed parents.

Although the precise mechanisms by which epigenetic modifications leads to TGP is not known, one possibility involves the transmission of DNA methylation^50,71^. Methylation of the glucocorticoid receptor (GR) and Fkbp5 (co-chaperone) appears to play a role in the transmission of predator stress effects to future generations. Female offspring from prenatal predator odor-exposed dams showed increased transcript abundance of both the GR gene and Fkbp5 in the amygdala^39^. Moreover, increased Fkbp5 expression was inversely correlated with decreased DNA methylation for this product’s gene^39^, a finding consistent with the human literature^26^. In a related study, female offspring of mice exposed to predator odor during pregnancy had decreased BDNF transcript abundance and a concomitant decrease in DNA methylation of BDNF exon IV in the hippocampus^40^. Epigenetic alterations of the BDNF gene are linked to impaired brain functioning, memory, stress, and neuropsychiatric disorders^72–74^. These results are consistent with other work in which predator scent stress induced the down-regulation of BDNF mRNA in the CA1 region of the hippocampus^75^, although more research is necessary to fully assess the role of DNA methylation in TGP.

While the impacts of TGP have been extensively explored in F1 individuals^21,58,63^, less research has addressed whether these effects can persist into the F2 generation. We found that F2 adult mice with at least one set of predator-stressed grandparents responded more strongly to a mild stressor, engaged in fewer social interactions, and exhibited increased anxiety-like behaviors than mice with only control grandparents. This result agrees with prior work on preconception^50,69,76^ and prenatal^77,78^ grandparental stress in lab rodents that found TGP can affect F2 behavioral phenotypes. While the F2 generation was not produced via cross-fostering, our data from the cross-fostered F1 experiment suggests that the F2 behavioral differences are similarly due to a biological mechanism. Because we cannot exclude the possibility of maternal behavior effects, however, future work assessing the impacts of predator stress in cross-fostered F2s is planned. A key difference between prior studies and the current work is that previous research assessed the impacts of stressors unrelated to predation risk on the F2 generation. While predator-induced grandparental TGP has been found in invertebrates^55,79^ and fish^80^, this appears to be its first confirmed occurrence in mammals.

Our experimental design also allowed us to parse out the relative influence of maternal versus paternal grandparent predation risk on the F2 phenotype. In general, we did not find a differential contribution from the grandmother or grandfather. Determining if there are different paternal and maternal grandparent contributions to TGP as well as grandmaternal and grandpaternal, e.g.,^80^ has been an increasingly active area of research^58^. Our results are in line with work assessing the TGP effect of chronic restraint stress on rats which found that both maternal and paternal grandparent experience had similar effects on F2s of both sexes^76^. Research using chronic unpredictable stressors, however, broadly concluded that paternal grandparent experience affected F2 female rats more than F2 males but that maternal grandparent experience had similar effects on both F2 sexes^69^. More work is clearly needed to understand when the differential grandparental contributions to TGP occur.

The results of our lab-based work have important ecological implications: even fleeting exposure to predation risk can affect the physiology and behavior of multiple subsequent generations. Research into predator-induced TGP effects generally employs repeated^38,80^ or chronic^46,55^ exposure to risk. While chronically high-stress situations can occur in the field, most prey are unlikely to survive, say, 60–100 h of imminent predator attack^54^. By contrast, our risk treatment (one five-minute exposure to a rat) almost certainly underestimates the predator threat perceived by most free-living prey. The fact that such a ‘low intensity’ encounter in the F0 generation nonetheless affected both F1 and F2 individuals suggests that the effects of predator-induced TGP may be ubiquitous in some—and perhaps most—prey individuals found in natural systems. An important caveat to this conclusion is that our research was carried out using lab-reared mice whose responses to predation risk may differ from those found in wild populations e.g.^81^. While the controlled conditions necessary to conduct our work would be challenging to maintain in the field, future research could address this possibility by starting with wild-caught F0 individuals. Furthermore, in the current set of experiments, animals are unable to escape the predator. However, in future studies, comparing the effects of environments in which prey can or cannot escape on subsequent brain and behavior will be helpful in teasing apart the contribution of controllability in the fear response. Finally, both parents were exposed in our experiment, which, although it would generally be the case in the wild when predation threat is high, may also have affected the results.

The traumatic effect of stressful events on an individual are well-known, and the ability of such stressors to affect future generations, through biological and/or social transmission, is increasingly recognized. In humans, these changes can make children and grandchildren more prone to mental illnesses such as anxiety, depression and/or posttraumatic stress disorder; in non-human systems their effects can be seen at the individual, population, and community scales. Our data illustrate that surprisingly, even a small amount of pre-conception stress both affects an individual and can alter the brain and behavioural responses of future generations. Moreover, based on our results, one can speculate that some degree of predator-induced epigenetic change may be the rule rather than the exception in natural systems. It is important to note, however, that our experiments were run on inbred laboratory animals, while the effects of acute pre-conception predator stress in a natural setting is not yet known. To fully understand the effects of predator stress on future generations, mechanistic and behavioral studies in wild animals following acute stressors must be completed.

The ‘long shadow’ of a single pre-conception exposure to predation risk raises the intriguing question: would acute exposure to other stressors have similar multi-generational impacts? Plants, for instance, respond differently to herbivory versus similar damage inflicted by mechanical wounding i.e., clipping a leaf with scissors^82^. From an informational perspective, grandparental encounters with predators may be a more reliable cue^45^ of future risk than electric shocks and other ‘unnatural’ stressors. Ultimately, identifying the mechanistic basis for and ubiquity of altered stress susceptibility in future generations will represent a major advance in several fields and may lead to novel treatments for devastating, and often treatment-resistant human neuropsychiatric disorders.

## Materials and methods

### Ethical approval

Protocols and procedures for all experiments were followed in accordance with the guidelines of the Canadian Council on Animal Care and approved by Memorial University of Newfoundland’s Animal Care Committee and in accordance with ARRIVE guideline.

### Animals

Male and female C57BL/6 mice were used in all experiments. All mice were given ad libitum access to food and water in standard laboratory conditions (i.e., temperature and humidity) on a 12-h light–dark cycle (lights on at 7:00 AM). Male Long-Evans rats (150–200 g in weight) were used as stimulus animals for the rat exposure. Rats were kept on a reverse light/dark cycle (lights off at 7:00 AM) and food restricted to 85% of expected body weight to increase activity and interaction rate with mice. Animals were purchased from Charles River Laboratories (St. Constant, QC, CA) and left undisturbed in their cages for at least one week after arrival prior to experimentation.

### General procedures

#### Predator stress

The exposure chamber was a standard plexiglass rat cage (47 cm × 26 cm × 20 cm) containing a clear plexiglass partition to divide the cage width into two compartments. Small holes in the partition allowed free olfactory flow. A piece of clear perforated plexiglass was placed on top of the cage to prevent animals from escaping or entering the opposite side of the cage. A mouse was exposed to a rat for either two or five minutes depending on the specific experiment (or part of the experiment). In a pilot experiment, we examined the effects of the 2 min exposure on mouse behavior (see supplementary methods 1.4).

Rats and mice were habituated to the exposure chamber once a day for the five days preceding exposure by placing the mouse or rat inside the cage for five minutes and allowing it to explore their side of the partitioned cage while the opposite side was unoccupied. We used two identical cages for habituation so that no mouse was habituated in a cage used to habituate a rat and vice versa. Mouse habituation always occurred before rat habituation, and the two species were never in the same or adjacent rooms until the day of exposure. On exposure day (day 6), the mouse was placed in the left side of the exposure chamber; the right side of the chamber contained either a live rat (Predator Stressed group) or was left empty (Control group). Control mice were run before predator exposed mice to reduce rat scent exposure. Following exposures, mice were returned to their home cages. All exposures were video recorded and hand-scored for mouse freezing duration and frequency (blind to group) as an index of fear and innate defensive behavior. Freezing was defined as immobility except for respiration. All chambers were wiped down with 70% ethanol between habituation trials and exposures.

#### F0s and F1s

Sexually inexperienced male and female C57BL/6 mice, aged 7–8 weeks, were randomly assigned to either the Predator Stressed (PS) or Control (C) groups. Following the 5-day habituation period, PS mice were exposed to a live rat in the exposure chamber for 5 min while C mice were exposed to an empty chamber. Two days after exposure, all mice were tested for anxiety-like behaviors in the elevated plus maze (EPM; described in supplementary methods 1.1). Ten days after exposure (8 days after the EPM), male and female C mice were bred together (n = 28 breeding pairs) and male and female PS mice were bred together (n = 29 breeding pairs). Breeding pairs were housed together for 7 days.

All F1s were left undisturbed with their mothers, except when ear notched for identification and cage cleaning, until weaning. F1s were weaned on approximately PND 21 and housed with same-sex littermates in groups no larger than five thereafter. There were no differences in litter size, number of litters, and % of male pups across groups (all p > 0.3).

### Experiments

#### 1A. Effects of preconception predator stress on F1 behavior

On PND 24, F1s (PS n = 61, C n = 41) underwent a six-test behavioral battery (one test per day for six days). The behavioral battery started with the EPM, followed by the open field (OF), light/dark box (LDB), acoustic startle test (AST), forced swim test (FST), and the social interaction test (SIT). Detailed descriptions of each test are provided in supplementary methods 1.1.

On PND 55, mice started the 5-day exposure habituation period. On PND 60, each mouse was subjected to a mild stressor: 2-min rat exposure. This was the first time F1 mice were exposed to a rat. On PND 62, all mice started a second six-day behavioral battery identical to the first one.

#### 1B

The adult behavioral battery was performed starting on PSD 62 on a separate group of F1s (PS n = 13, C n = 14) that did not undergo the 5-day habituation or the 2-min rat exposure.

#### Physiological and molecular effects of predator stress

Experiment 2 assessed neuronal activity (cFOS) in the hippocampus and glucocorticoid system function (plasma corticosterone). Only males were used for these assays as there were no sex differences found in the behavioral measures. To obtain the tissue, transcardial perfusion was used to maintain tissue, using Urethane (15%, prepared in distilled water) as an anesthetic. For euthanasia, the animals were perfused with ice cold 4% paraformaldehyde (PFA; Fisher Scientific, Hampton, New Hampshire, USA; in 0.1 M phosphate buffer, pH 7.4), after a 1-min saline (0.9%) pre-flush to remove all blood. The brains were removed from the skull and post-fixed individually, in a 4% PFA solution. Twenty-four hours prior to slicing, brains were immersed in 20% sucrose. All brains were stored in a 4 °C environment, with all solutions at ice cold temperatures to prevent the melting of tissues. Procedures for the hormone assay and immunostaining are detailed in supplementary methods 1.2 and 1.3.

Corticosterone: physiological analyses were run on two sets of mice. The first set was 8- to 10-week-old male mice after a 5-min rat (PS n = 5) or control (C n = 5) exposure. The second set was male PS F1 (n = 5) and C F1 (n = 5) mice (offspring from the breeding pairs described in experiment 1) after a ‘two-minute rat exposure’ or ‘no rat exposure’ on PND 60.

Thirty minutes after the stressor exposure, mice were euthanized and 500 μl of trunk blood collected. Blood was also collected in F1s that that did not undergo the rat exposure on PND 60. Blood was processed and quantified for corticosterone levels using an ELISA (n = 5 mice/group in both experiments).cFOS: molecular and histological analyses were run on two sets of mice. The first set was 8- to 10-week-old male mice after a five-minute rat (PS n = 4) or control (C n = 4) exposure. The second set was male PS F1 (n = 4) and C F1 (n = 4) mice (offspring from the breeding pairs described in experiment 1) after a ‘two-minute rat exposure’ on PND 60 (Fig. 4A).

Ninety minutes after the stressor exposure, mice were anesthetized and perfused. Their brains were then extracted, sectioned, processed, and quantified for cFOS detection in chromogenic immunohistochemically stained sections.

#### Effect of maternal social environment on F1 behavior

Experiment 3 followed the same protocol as experiment 1 except for the fostering procedure. Fostering was initiated 3–4 h after parturition. The biological mother was removed from each litter, the litter thoroughly mixed with the foster mother’s bedding, and the litter plus bedding placed in a clean warm cage. All mice from a litter were placed with the same foster mother. Foster mothers were observed for at least ten min to ensure acceptance of the new litter. All F1s were fostered to either a novel C or PS mother, creating four groups: PS F1s fostered to PS mother (PS biological parents-PS foster mother (‘BS-FS’, n = 30), PS F1s fostered to C mother (‘BS-FC’, n = 23), C F1s fostered to PS mother (‘BC-FS’, n = 23), and C F1s fostered to C mother (‘BC-FC’, n = 34). All F1s were weaned at PND 21 and underwent the behavioral test battery on PND 24–30 (supplementary methods 1.1). They were then exposed to the mild stressor (2 min RET) at PND 60 and underwent a second behavioral test battery on PND 62–68.

#### Effects of F0 preconception predator stress on F2 behavior

In experiment 4, the F1 parents of the F2 generation were generated as described in experiment 1, but at weaning the F1s used for this experiment underwent neither the behavioral test batteries nor the mild stressor and were instead left undisturbed. These F1s (n = 9 total breeding pairs) were used to generate four groups of F2 mice (GFC: grandfather control, GMC: grandmother control, GFPS: grandfather predator stressed, GMPS: grandmother predator stressed). F2 mice were weaned and separated on PND 21 and began the behavioral battery on PND 24 (supplementary methods 1.1). The four F2 groups [GFC-GMC (n = 24), GFC-GMS (n = 20), GFS-GMC (n = 14), and GFS-GMS (n = 21)] were habituated to the rat exposure chamber once a day for five days (PND 54–59), exposed to the mild stressor (two-minute rat exposure) on PND 60, and underwent a second behavioral test battery from PND 62–68.

### Statistical analysis

For the F0 generation, we used general linear models (GLM) with the fixed main effects of treatment (control, predator stressed) and sex (male, female) to analyze behavior, CORT, and cFOS expression. For the F1 and F2 generations, we used GLMM (normal distribution with link identity function) with the appropriate main treatment effects, offspring sex, and offspring litter coded as a random effect. For the histology data we used the Benjamini–Hochberg procedure to control for false discoveries when comparing stressed and control animals^83^. Differences between groups (control vs stressed) were considered significant at p < 0.05.

## Supplementary Information

Supplementary Information.

## Data Availability

The datasets used and/or analysed during the current study available from the corresponding author on reasonable request.
