# Supplementary material for: A short pre-conception bout of predation risk affects both children and grandchildren
Source: Sci Rep. 2023 Jul 5;13:10886. doi: 10.1038/s41598-023-37455-9 (PMC10322924; doi:10.1038/s41598-023-37455-9)

Full title: A short pre-conception bout of predation risk affects both children and grandchildren

Corresponding author: Jacqueline Blundell

Supplementary Methods

SUP 1.1 Behavioral tests

Elevated plus maze (EPM): The EPM was made of white plexiglass (0.6 cm thick) and consisted of four 29.0 long x 5.1 cm wide arms with a square, 10.2 cm^2 center platform connecting the arms at right angles. The two closed arms were on opposite sides of the platform and enclosed by three 14 cm walls that left the ceiling and platform entrance open. The two open arms had neither walls nor a ceiling but had a 0.5 cm lip. The maze was elevated by four 45 cm legs positioned at the end of each arm. Mice were placed facing away from the center platform and allowed to freely explore the maze for five minutes. Time in open and closed arms and number of entries into open closed arms was calculated with the EthoVision XT10 tracking system (Noldus, Wageningen, NL). Ratio time was calculated as time in open arms divided by time in all arms. Ratio frequency was calculated as number of entries into open arms divided by number of entries in all arms.

Open field (OF): The OF was carried out in a 48.0 x 48.0 x 48.0 cm grey plexiglass box. A square was marked off by tape on the floor of the box 10 cm from each wall to determine time spent in the box center and perimeter. Mice were placed in the center of the box and allowed to freely move around the arena for five minutes. Duration in the center and distance traveled were calculated with EthoVision

Light/dark box (LDB): The LDB arena was made from two 20.3 x 20.3 x 14.9 cm grey plexiglass boxes connected by a 10.2 x 6.4 x 7.6 cm corridor. The light box had a clear plexiglass removable lid with 25 ventilation holes; the dark box had an opaque grey plexiglass removable lid. A nine W, 550 lm light was positioned 5.1 cm above the light box. Mice were placed into the light box and allowed to freely move in the arena for five minutes. Video recordings were taken from above the light box for later analysis. The number of entries into and total time in the light side of the box was measured from video by a researcher blind to treatment.

Acoustic startle test (AST): Mice were placed in a 12.7 cm long x 3.7 cm wide cylindrical animal enclosure. An electric transducer below the enclosure recorded activity throughout the experiment. All enclosures were placed inside a San Diego Instruments (San Diego, CA, USA) startle chamber. Mice were habituated to a background white noise of 50 DB for five minutes before receiving 120 DB pulses of white noise every 30 seconds for 15 minutes. Peak startle amplitude was determined by subtracting the transducer output at the beginning of the noise burst (Vstart) from the maximum transducer output (Vmax) during the recording window.

Force swim test (FST): A 66.0 cm tall x 24.5 cm wide cylindrical plexiglass container was filled with water (22 ± 1 °C temperature) to a height of approximately 15 cm. Mice were placed into the pool and allowed to swim freely for six minutes. The first two minutes were used to allow mice to habituate to the water and not used for analysis. Video recordings were taken from the front of the cylinder and the total amount of time the mice spent immobile and the number of times the mice were immobile was scored by a researcher blind to treatment.

Social interaction test (SIT): The SIT took place in the OF arena. In the first 150 seconds, an empty cage with metal bars was placed against the center of one wall with the subject mouse

free to travel around the arena. The subject was then briefly removed and a novel male C57BL6 mouse placed inside the metal cage. The subject was then returned to the arena for an additional 150 seconds. Video recordings were taken from above and scored by a researcher blind to treatment. Ratios were calculated to determine the frequency and amount of time spent interacting with the empty cage compared to when the cage contained a novel conspecific.

SUP 1.2 Hormone assay

Corticosterone concentrations were measured using a commercial ELISA kit (Arbor Assays Company, Ann Arbor, MI, US) with a minimum detection level of 16.9 pg/ml corticosterone. The kit measured total corticosterone in serum including the corticosterone combined with corticosteroid-binding globulin (CBG). The cross reactivity of the kit for cortisol was 0.38% (tested at 50% binding). Briefly, 50 µl of standards or samples were added in duplicate to wells of the microtiter plate. Assay buffer (75 µl) was added to the non-specific binding (NSB) wells and 50 µl of assay buffer was added to wells to act as maximum binding wells. Then, 25 µl of the DetectX Corticosterone Conjugate and 25 µl of the DetectX Corticosterone antibody (except the NSB wells) were added to each well and the titer plate was shaken for 1 h at room temperature. After the plate was washed using the wash solution and blot dried, 100 µl of tetramethylbenzidine (TMB) substrate was added to each well and incubated for 30 min at room temperature. The optical density (OD) of corticosterone was read at 450 nm wavelength using a plate reader within 15 minutes of the reaction being terminated by adding 50 µl of the stop solution. Corticosterone concentration was calculated using standard curves.

SUP 1.3 Immunostaining for F0 and F1 generation mice

Brain collection for immunostaining: Brain collection occurred 90 minutes (for c-FOS; [1,2]), or two days (GR, FKBP5) after exposure [3,4]. Animals were anaesthetized with 15% urethane prepared in distilled water and, after a 1-minute saline (0.9%) pre-flush to remove all blood, transcardial perfused with ice-cold 4% paraformaldehyde (PFA in 0.1M phosphate buffer, pH 7.4). Brains were then removed and individually post-fixed in a 4% PFA solution. One day before sectioning, brains were immersed in 20% sucrose. Brains were stored at 4°C and all solutions were ice-cold to prevent tissue melting.

Slide preparation for analysis: A Leica CM3050 S cryostat machine (Leica Biosystems, Wetzlar, DE) was used to section all brain samples with a D-profile tungsten knife. The cryostat specimen temperature was set to -19 ± 3°C and the chamber temperature set to -17 ± 3°C. All slicing was conducted manually, with section and trimming thickness set to 30µm for all sections. Coronal sections were collected, prioritizing the cortex, ventricles, and hippocampus. All slides were transferred to slide storage boxes and kept in a -80°C environment until staining.

Immunohistochemistry for cFOS: Immunostaining followed previously published procedures [5]. During staining, slides were removed from the -80°C storage and thawed at 4°C for 10 minutes, followed by eight minutes at room temperature to further dry. Slide borders were marked with a PAP hydrophobic pen to create an antibody barrier. The primary antibody, rabbit cFOS (1/1000, Cell Signaling, Danvers, MA, US), was diluted in phosphate buffered saline (PBS) with PBS + 0.2% TritonX-100 and 2% normal goat serum. One mL of primary antibody was applied to each slide and incubated for 48 hours at 4°C. Slides were then washed for 20 minutes with PBS before being incubated in the secondary antibody (biotinylated goat anti-rabbit diluted in PBS, 0.2%triton x-100 and 2% normal goat serum) for two hours. Washing procedures were repeated for 20 minutes before incubation in Vectastain avidin/biotinylated enzyme (A+B)

solution for one hour (Vector Labs, Burlingame, CA, US). Washing was repeated and the reaction product visualized by adding 0.05% 3,3'-Diaminobenzidine (DAB) with 0.01% hydrogen peroxide in distilled water for five minutes. Sections were then washed with distilled water and air dried for 24 hours before being dehydrated with alcohol/xylene and cover slipped with Permount (Thermo Fisher Scientific, Waltham, MA, US).

c-FOS staining analysis: Images of sections were captured with a digital camera (Teledyne QImaging, Surry, BC, CA) connected to a bright field microscope (Olympus Life Science, Tokyo, JP) at 10x, 20x and 40x magnifications. Olympus cell Sens imaging software was used to process and store images. Representative fields consisted of eight subregions within the hippocampus (dentate gyrus and Cornu Ammonis 1- CA1) and two sub-regions (central and basolateral) within the amygdala. Within the dentate gyrus, we measured cFOS in the dorsal right hemisphere (DENTRH), dorsal left hemisphere (DENTLH), ventral right hemisphere (VDRH), and ventral left hemisphere (VDLH). Within the CA1 area, we measured cFOS in the dorsal right hemisphere (CA1RH), dorsal left hemisphere (CA1LH), ventral right hemisphere (VCA1RH), and ventral left hemisphere (VCA1LH). All sections were analyzed at a constant microscope light intensity. The intensity of cFOS staining was normalized with background OD and analyzed using ImageJ (National Institute of Health (NIH), Bethesda, MD, US).

SUP 1.4 SUP Experiment 1: Effect of two-minute rat exposure on mouse behavior

Sexually inexperienced male C57BL/6 mice, aged 7-8 weeks, were divided into two groups: PS (n=8) and C (n=8). All mice were habituated to the exposure chamber for five minutes per day for five consecutive days. On day six, mice in the PS group were exposed to a live rat in the exposure chamber for two minutes, while mice in the C group were exposed to an empty chamber for two min. Starting on day eight, all mice underwent a six-test behavioral battery (one test per day for six days). The behavioral battery started with the elevated plus maze, followed by the open field, light/dark box, acoustic startle test, forced swim test, and the social interaction test. Detailed descriptions of each test are provided in supplementary methods 1.1.

Supplementary Results

SUP 2.1 *Two minute exposure to a rat does not increase subsequent anxiety- or depressive-like behavior.*

As expected, mice exposed to a rat froze more often than mice exposed to an empty cage ($F_{1,14}$=6.669, p=0.022]. Across all behavioral tests, there were no significant differences in the elevated plus maze, light/dark box, startle, forced swim test or social interaction test. There was a significant treatment effect on time in the center of the open field [$F_{1,14}$=6.754, p=0.02]. Surprisingly, predator stressed mice spent more time in the center than control mice.

Literature Cited

1 Bullitt, E. Expression of C-*fos*-like protein as a marker for neuronal activity following noxious stimulation in the rat. *J. Comp. Neurol.* **296**, 517-530, doi:10.1002/cne.902960402 (1990).

2 Figueiredo, H. F., Bodie, B. L., Tauchi, M., Dolgas, C. M. & Herman, J. P. Stress integration after acute and chronic predator stress: differential activation of central stress

circuitry and sensitization of the hypothalamo-pituitary-adrenocortical axis. *Endocrinology* **144**, 5249-5258, doi:10.1210/en.2003-0713 (2003).

3 Asada, M. *et al.* DNA binding-dependent glucocorticoid receptor activity promotes adipogenesis via Kruppel-like factor 15 gene expression. *Lab. Investig.* **91**, 203-215, doi:10.1038/labinvest.2010.170 (2011).

4 Garrett, L. *et al.* Conditional reduction of adult born doublecortin-positive neurons reversibly impairs selective behaviors. *Front. Behav. Neurosci.* **9**, 302, doi:10.3389/fnbeh.2015.00302 (2015).

5 Bhattacharya, S. *et al.* Histone deacetylase inhibition induces odor preference memory extension and maintains enhanced AMPA receptor expression in the rat pup model. *Learn. Mem.* **24**, 543-551, doi:10.1101/lm.045799.117 (2017).

Supplementary Figure Legends

Supplementary Figure 1. Predator stress increases plasma corticosterone levels. Mean ± SEM plotted over two groups: control and predator stress. Plasma corticosterone levels were higher in predator stressed mice compared to controls. * $p<0.05$

Supplementary Figure 2. Predator stress increases cFOS expression. cFOS expression was measured in the hippocampus within the dorsal dentate gyrus, left and right hemisphere (DENTRH, DENTLH), ventral dentate gyrus, left and right hemisphere (VDLH, VDRH), dorsal CA1, left and right hemisphere (CA1LH, CA1RH), and ventral CA1, left and right hemisphere (VCA1LH, VCA1RH). Panel C: Mean ± SEM cFOS expression in different brain regions. Excluding CA1 LH, expression was elevated in the dentate and CA1 in predator-stressed mice compared to controls. * $p<0.05$, ** $p<0.01$, *** $p<0.001$, NS: not significant

Supplementary Figure 3. Preconception predator stress produces anxiety-like behaviour in adolescent F1 mice. Panels A-F: Mean ± SEM for four groups of adolescent F1 offspring: males and females from control parents (Control F1♂ and ♀), and males and females from predator-stressed parents (Predator stressed F1♂ and ♀). F1 offspring of predator-stressed parents spent less time in the open arms (ratio time, Panel A) and entered the open arms less often (ratio frequency, Panel B) in the elevated plus maze (EPM), spent less time in the center (Panel C) and travelled less distance (Panel D) in the open field, interacted less (social interaction ratio, Panel E) in the social interaction test, and had a higher peak startle amplitude (Panel F) in the acoustic startle test compared to control F1s. * $p<0.05$, ** $p<0.01$, *** $p<0.001$

Supplementary Figure 4. Preconception predator stressed F1 mice show increased anxiety-like behaviour in the absence of mild stress exposure. Mean + SEM for two groups of F1 offspring: those from control parents (Control F1) and those from predator-stressed parents (Predator stressed F1). Offspring of predator-stressed parents spent less time (Panel A) in the open arms and entered (Panel B) the open arms of the elevated plus maze less often than control F1s. Predator stressed F1s travelled less distance (Panel C) and tended to spend less time in the center (Panel D) of the open field, spent less time in the light side (Panel E) of the light/dark box, and had a higher peak startle amplitude (Panel F) in the acoustic startle test compared to control F1s. * $p<0.05$, ** $p<0.01$, NS: not significant

Supplementary Figure 5. Biological parent stress experience, not social environment, determines anxiety-like behaviour and hyperarousal in adolescent F1 mice. Mean ± SEM plotted over four groups: offspring of control biological parents that were cross-fostered to a control mother, offspring of control biological parents that were cross-fostered to a

predator-stressed mother, offspring of predator-stressed biological parents that were cross-fostered to a control mother, and offspring of predator-stressed biological parents that were cross-fostered to a predator-stressed mother. The offspring of predator-stressed biological parents, regardless of cross-fostering mother, spent less time in the open arms (ratio time, Panel A) and entered the open arms less often (ratio frequency, Panel B) in the elevated plus maze, entered the light side less often (Panel C) in the light/dark box, spent less time in the center (Panel D) and travelled more distance (Panel E) in the open field, and had a higher peak startle amplitude (Panel F) compared to offspring of control biological parents, regardless of cross-fostering mother. * $p<0.05$, ** $p<0.01$, NS: not significant

Supplementary Figure 6. Preconception predator stress increased anxiety-like behaviour in second filial (F2) adolescent mice. Mean $\pm$ SEM plotted over four groups: two control grandparents (GFC-GMC), control grandfather and predator-stressed grandmother (GFC-GMS), predator-stressed grandfather and control grandmother (GFS-GMC), and two predator-stressed grandparents (GFS-GMS). F2s with at least one predator-stressed grandparent spent less time in the open arms (ratio time, Panel A) and entered the open arms less often (ratio frequency, Panel B) in the elevated plus maze, travelled less distance (Panel C) and spent less time in the center (Panel D) in the open field. * $p<0.05$, *** $p<0.001$

Supplementary Figure 1.

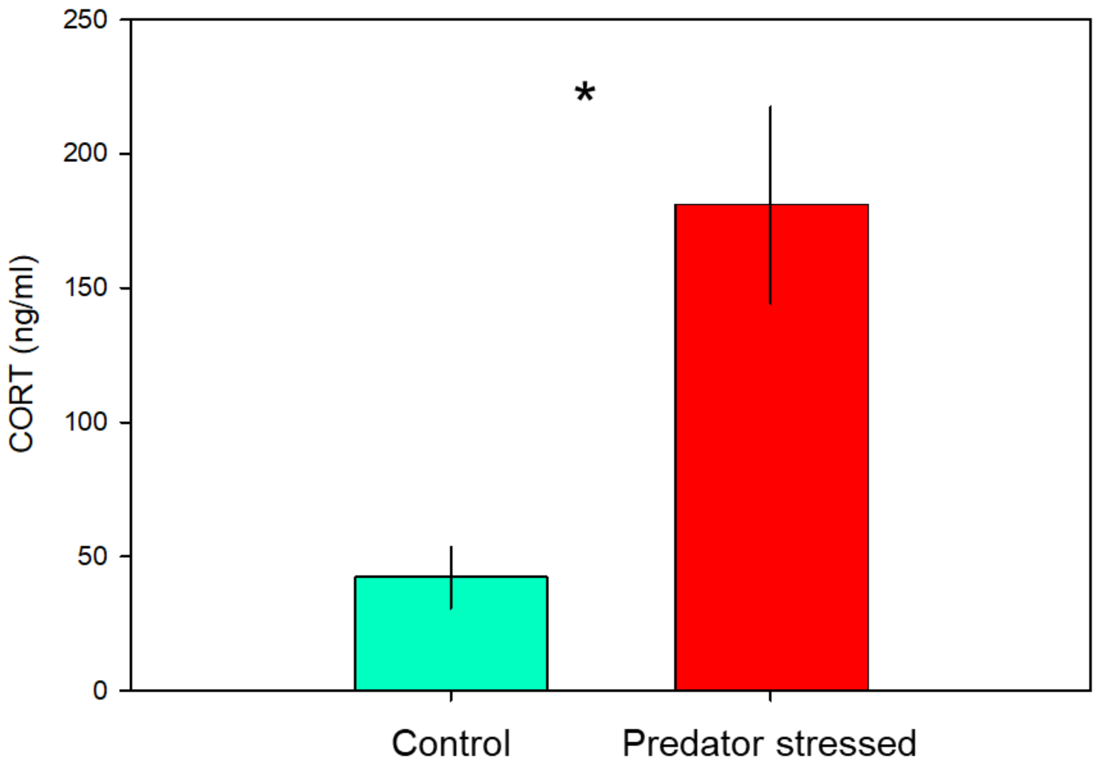

Supplementary Figure 2.

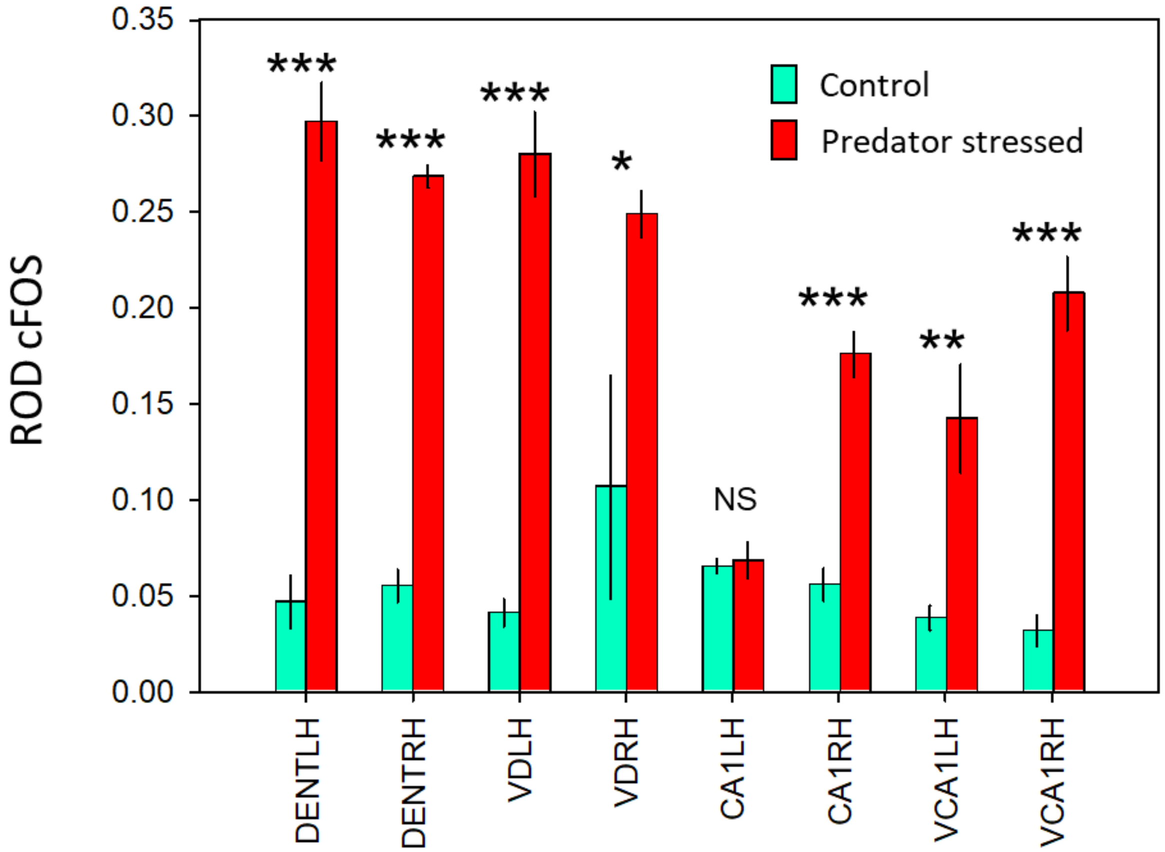

Supplementary Figure 3.

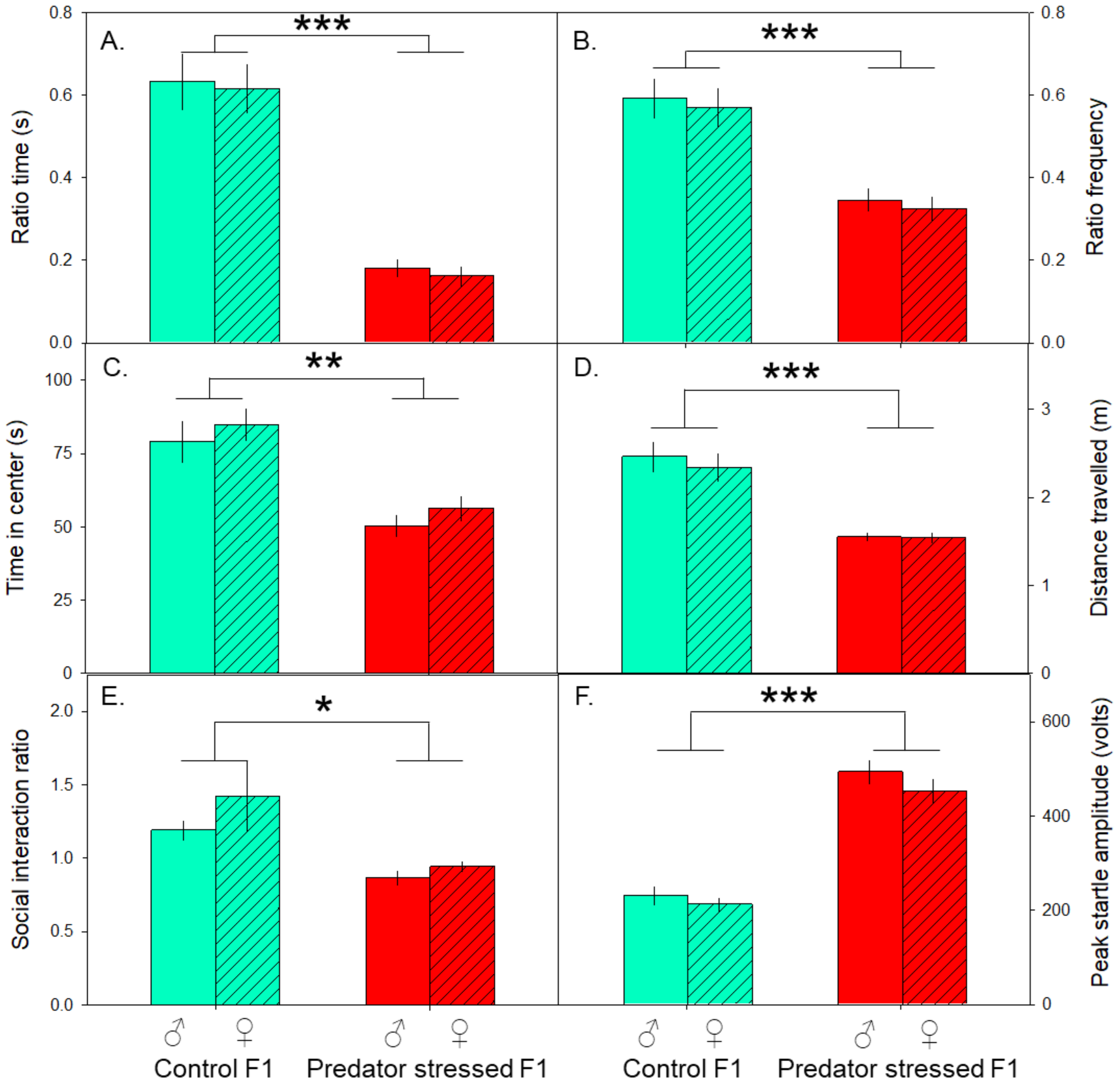

Supplementary Figure 4.

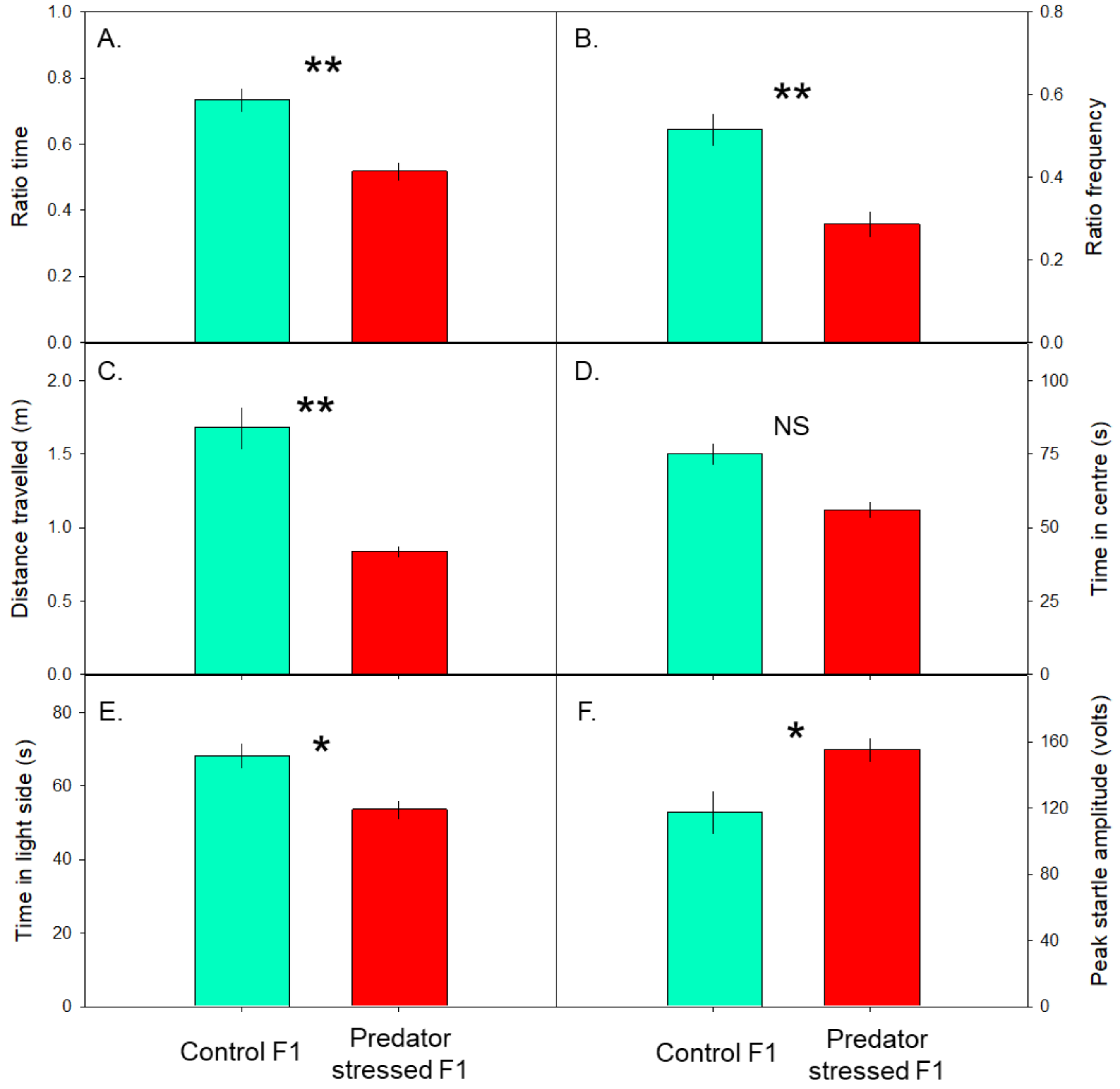

Supplementary Figure 5.

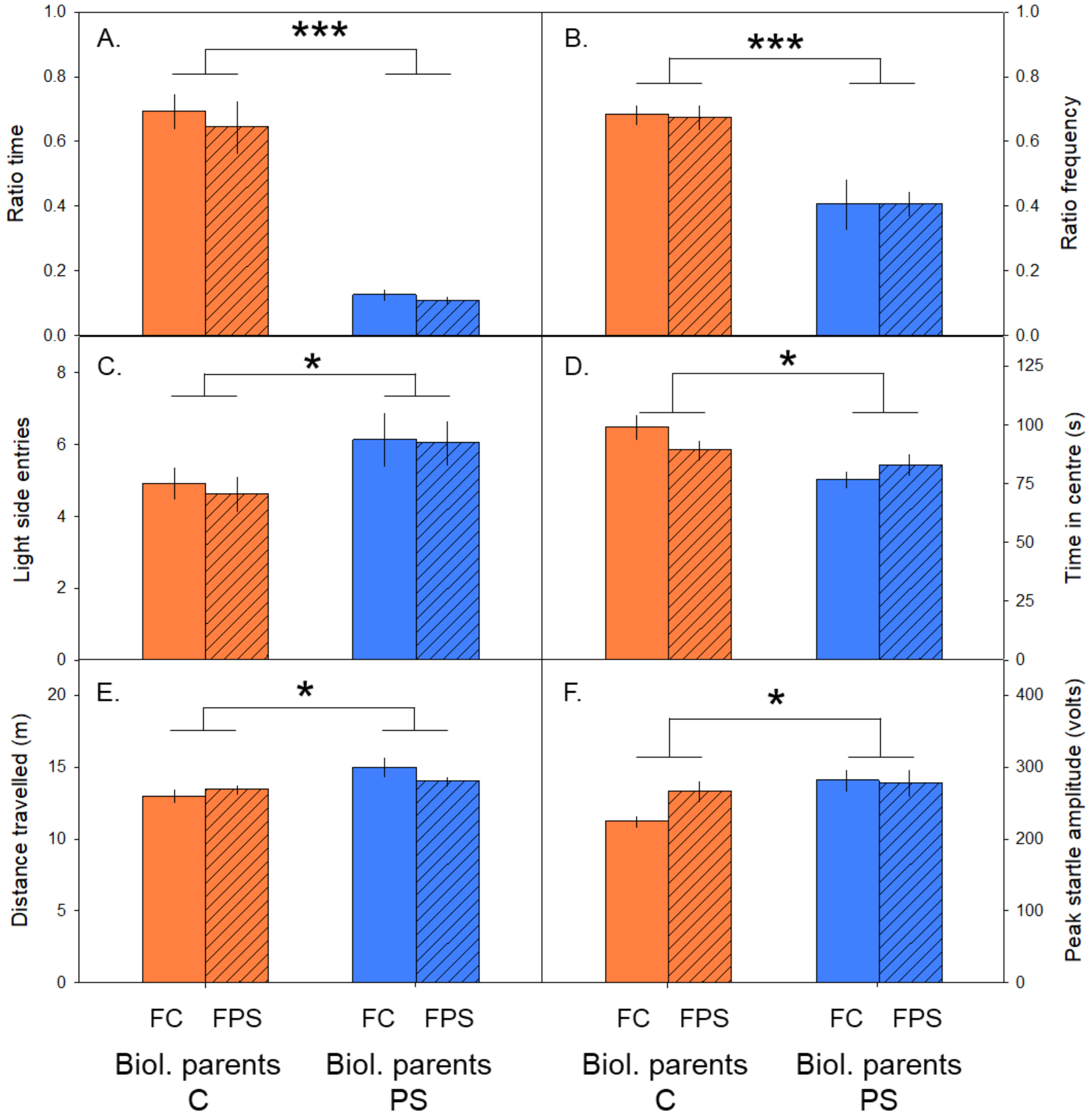

Supplementary Figure 6.

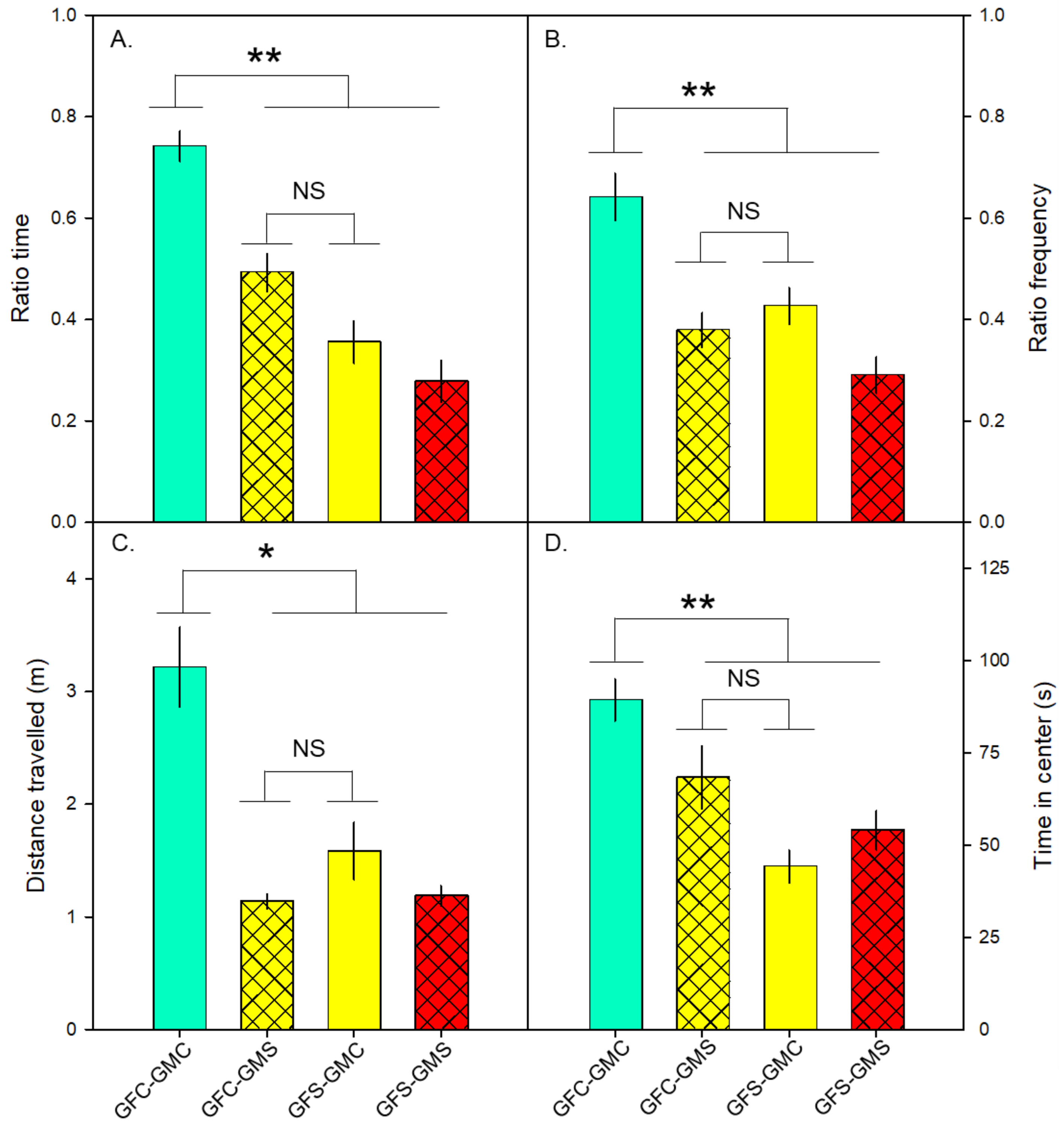

Supplement: Supplementary file 1 — Supplementary Information. [file 41598_2023_37455_MOESM1_ESM.pdf]
